# A combination of cytokeratin 5/6, p63, p40 and MUC5AC are useful for distinguishing squamous cell carcinoma from adenocarcinoma of the cervix

**DOI:** 10.1186/s13000-020-01018-7

**Published:** 2020-08-26

**Authors:** Hailing Li, Xiaotong Jing, Jie Yu, Jiannan Liu, Tingguo Zhang, Shiming Chen, Xiaofang Zhang

**Affiliations:** 1 Department of Pathology, Weifang Traditional Chinese Hospital, Weifang, Shandong P. R. China; 2 Department of Pathology, School of basic Medical Science; Shandong University, Jinan, Shandong P. R. China; 3 Department of Pathology, the Fourth Hospital of Jinan & the third affiliated hospital of Shandong first medical university, Jinan, Shandong P. R. China; 4 Department of Oncology, Yuhuangding Hospital, Yantai, Shandong P. R. China; 5 Department of Pathology, School of basic Medical Science, Shandong University, Jinan, 250012 Shandong P. R. China

**Keywords:** Cervical adenocarcinoma, Cervical squamous cell carcinoma, MUC5AC, CK7

## Abstract

**Purpose:**

Squamous cell carcinomas and adenocarcinomas are the most common types of cervical cancer. Compared to squamous cell carcinomas, adenocarcinomas are more common in younger women and have a poorer prognosis. Yet, so far, no useful biomarkers have been developed for these two types of cancer. In the following study, we examined the combination of cytokeratin 5/6, p63, p40 and MUC5AC for distinguishing squamous cell carcinoma (SCC) from adenocarcinoma of the cervix (AEC).

**Materials and methods:**

A total of 101 SCC and 108 AEC were collected. Immunohistochemical analyses were conducted to determine the expression of CK5/6, p63, p40, CK7 and MUC5AC. One pathologist who was blinded to the patient’s clinical and pathological data interpreted the staining results.

**Results:**

MUC5AC and CK7 were detected in 81.48 and 82.41% of AEC cases compared to 9.9 and 49.50% of SCC cases (*P* < 0.05); the specificity of MUC5AC was higher than that of CK7 in AEC (P < 0.05). The sensitivity of MUC5AC combined with p40 or p63 was similar to that of CK7, but the specificity was slightly higher than that of CK7 in AEC. Moreover, the expression of MUC5AC was correlated with the degree of tumor differentiation in adenocarcinomas (*P* = 0.036) and was not related to the prognosis of cervical adenocarcinoma and subtypes.

**Conclusions:**

MUC5AC may be useful as a biomarker for differential diagnoses between squamous carcinoma and adenocarcinoma of the cervix.

## Introduction

Cervical cancer is the fourth most common carcinoma in women responsible for 10–15% of cancer-related deaths worldwide [1, 2]. Squamous carcinoma is the most common type of cervical carcinoma, followed by adenocarcinoma. Nevertheless, over the last three decades, a significant increase in adenocarcinoma cases has been observed in many developed countries, especially in younger women [3]. Pap-smear screening, also known as Pap test, is still considered the main screening method for cervical cancer, especially for squamous carcinoma [4]. Compared to squamous carcinoma, the adenocarcinoma of the cervix is more common in younger women and has a poorer prognosis [5]. Therapeutic approaches include chemo-radiotherapy (CCRT), which has been proven to be effective for squamous carcinoma of the cervix, but not for adenocarcinoma of the cervix [6], due to its high chemo- and radio-resistance [7]. Therefore, differentiating adenocarcinoma from squamous carcinoma is important in order to provide patients with most suitable therapy.

p63, p40, and cytokeratin 5/6(CK5/6) are the most common panel of immunochemical markers for the diagnosis of squamous carcinoma [8]. p63 and CK5/6 are traditional markers that indicate squamous differentiation [9]. In primary lung neoplasms, most squamous carcinomas and large cell carcinomas are positive for CK5/6 [10]. Warth et al found that the probability of a correct SQCC diagnosis using CK5/6 is 86.9% [11].

p63, a transcriptional regulator, has a crucial role in the development and differentiation of stratified squamous epithelium. It is usually strongly expressed in the basal keratinocytes [12–14]. Vosmik et al analyzed 70 patients with cervical squamous cell carcinoma and found that 94.29% (66/70) had positive expression of p63 [15].

p40 is a new specific marker for distinguishing squamous carcinomas from adenocarcinoma, whose specificity is about 100% in lung carcinomas. However, the positive expression of CK5/6, p63, and p40 are only found in a few adenocarcinomas [10, 16]. Kriegsmann et al suggested the use of either CK5/6 or p40 over p63 in the routine diagnostic setting [17]. CK7 is expressed in many ductal and glandular epithelial cells (mainly gallbladder, hepatic ducts, and pancreatic ducts), in tissues of the female genital tract (ovary, endometrium, fallopian tube, and cervix), and in the breast, lung, and urinary tract tissues [18]. In the normal cervical tissue and adenocarcinoma, CK7 staining was observed in the columnar cells of endocervical glands. Hashiguchi et al found the different rates of CK7 in patients with cervical intraepithelial neoplasia and those with invasive carcinomas (96.7% vs. 72.9%) [19, 20]. Thus far, no efficient markers have been developed for distinguishing squamous cell carcinoma and adenocarcinoma in the endocervix.

Mucins are a family of large glycoproteins expressed on the epithelial cell surfaces, including ducts of lacrimal glands in the eye, salivary glands, the lining of the respiratory, gastrointestinal, urothelial and reproductive tracts [21]. MUC5AC belongs to gel-forming mucins [22]. Multiple histological studies have highlighted that MUC5AC is expressed in the conjunctiva, middle ear, nasopharynx, lungs, gallbladder, and stomach under normal conditions where it provides protection to corresponding epithelial surfaces from different factors [23]. Some research has shown that MUC5AC may be a potential biomarker in pancreatic cancer tissues [24]. DiMaio et al. found that anterior gradient homolog 2 and MUC5AC are useful positive markers of adenocarcinoma in the setting of absent or diminished p63 and cytokeratin 5/6 staining in esophageal carcinoma [25]. It is also expressed in the endocervix. Yamanoi et al. found that MUC5AC was largely expressed in typical LEGH, atypical LEGH, GAS-MDA, and GAS-nonMDA [26]. Thus, we speculated that MUC5AC could be expressed in other adenocarcinomas and might be used for the differential diagnosis of adenocarcinoma and squamous carcinoma. The aim of this study was to examine the combination of cytokeratin 5/6, p63, p40, and MUC5AC for distinguishing squamous cell carcinoma (SCC) from the adenocarcinoma in the cervix (AEC).

## Materials and methods

### Tissue samples

We analyzed 101 poorly to moderately differentiated cervical squamous carcinoma (SCC) and 108 adenocarcinomas of endocervix (AEC). All tissues were collected from the Department of Human Pathology of Qilu Hospital, Shandong University, China, from 2008 to 2017. Specimens were retrieved from the pathology files of the Department of Pathology at the same hospital. After collection, all specimens were fixed in 10% buffered formalin. Hematoxylin & eosin (H&E) stains were available for review; paraffin blocks were used for immunohistochemical staining. All the slides were reviewed by two experienced pathologists.

Histopathological and clinical variables, including age, tumor size, differentiation, infiltrate depth, and lymph node metastasis, were summarized in Table 1. Follow-up information was available in 91 AEC, with the follow-up time ranging from 8 to 90 months (mean 42.34 months).

**Table 1:** Comparison of clinicopathological features between cervical squamous cell carcinoma and cervical adenocarcinoma

	squamous cell carcinomas(*n* = 101)	adenocarcinoma(*n* = 108)	χ 2	*P* Value
**Age**				
≤ 45	49 (48.51%)	50 (46.30%)	0.103	0.748
> 45	52 (51.49%)	58 (53.70%)		
**Size (cm)**				
< 4	66 (65.35%)	75 (69.44%)	0.405	0.817
≥ 4	32 (31.68%)	30 (27.78%)		
unknown	3 (2.97%)	3 (2.78%)		
**Differentiation**				
Poor	101 (100.00%)	36 (33.33%)	102.720	< 0.001*
Moderate	0 (0.00%)	41 (37.96%)		
Well	0 (0.00%)	26 (24.07%)		
unknown	0 (0.00%)	5 (4.63%)		
**Infiltrate depth of mesenchyme**				
≤ 1/2	23 (22.77%)	43 (39.81%)	7.078	0.029*
> 1/2	75 (74.26%)	63 (58.33%)		
unknown	3 (2.97%)	2 (1.85%)		
**Lymph node metastasis**				
No	66 (65.35%)	74 (68.52%)	15.136	0.001*
Yes	34 (33.66%)	20 (18.52%)		
unknown	1 (0.99%)	14 (12.96%)		

### Immunohistochemistry

Four to five micron-thick paraffin sections of the 209 cases were dewaxed, rehydrated in graded alcohols, and processed using the PV-9000 detection kit (Zsbio Commerce store, Beijing, China). Briefly, antigen retrieval was performed in a microwave oven for 3 min in 10 mM Tris-EDTA buffer (10 mM Tris Base, 1 mM EDTA Solution, 0.05% Tween 20, pH 9.0). Endogenous peroxidase activity was blocked with a 1.7% H2O2-methanol solution for 30 min. Slides were then incubated in 10% normal goat serum for 30 min to prevent non-specific binding. Samples were then incubated overnight at 4 °C with a primary antibody. Phosphate Buffered Saline (PBS) was used instead of the first antibody as a negative control. Consequently, samples were incubated with Reagent 2 at room temperature for 30 min and Reagent 3 at room temperature for 20 min. Finally, the tissues were stained with diaminobenzidine (DAB). The antibodies used in this study are listed in Table 2.

**Table 2:** Immunohistochemical antibodies

Antibody	No.	Vendor	Diluation
MUC5AC	ZM-0395	Zsbio Commerce store, Beijing, China	Ready to use
CK5/6	ZM-0313	Zsbio Commerce store, Beijing, China	Ready to use
CK7	ZM-0071	Zsbio Commerce store, Beijing, China	Ready to use
p40	ZM-0472	Zsbio Commerce store, Beijing, China	Ready to use
p63	ZM-0406	Zsbio Commerce store, Beijing, China	Ready to use

### Scoring method

Staining results were interpreted by one pathologist who was blinded to the patient’s clinical and pathological data. For CK5/6, CK7, and MUC5AC, more than 5% of tumor cells with a membrane or cytoplasmic brown-yellow granules were considered positive. For p63 and p40, the positive standard was that more than 5% of tumor cells have brown-yellow granules in the nucleus.

### Statistical analysis

Statistical analysis was performed with SPSS software (Version 21.0, SPSS Inc. Chicago, II, U.S.A.). Chi-square or Fisher’s exact tests were used when comparing frequencies between two groups. Probability values less than 0.05 were considered statistically significant.

## Results

### The expression of CK5/6, p63, p40, CK7, and MUC5AC in SCC and AEC

**Fig. 1:**
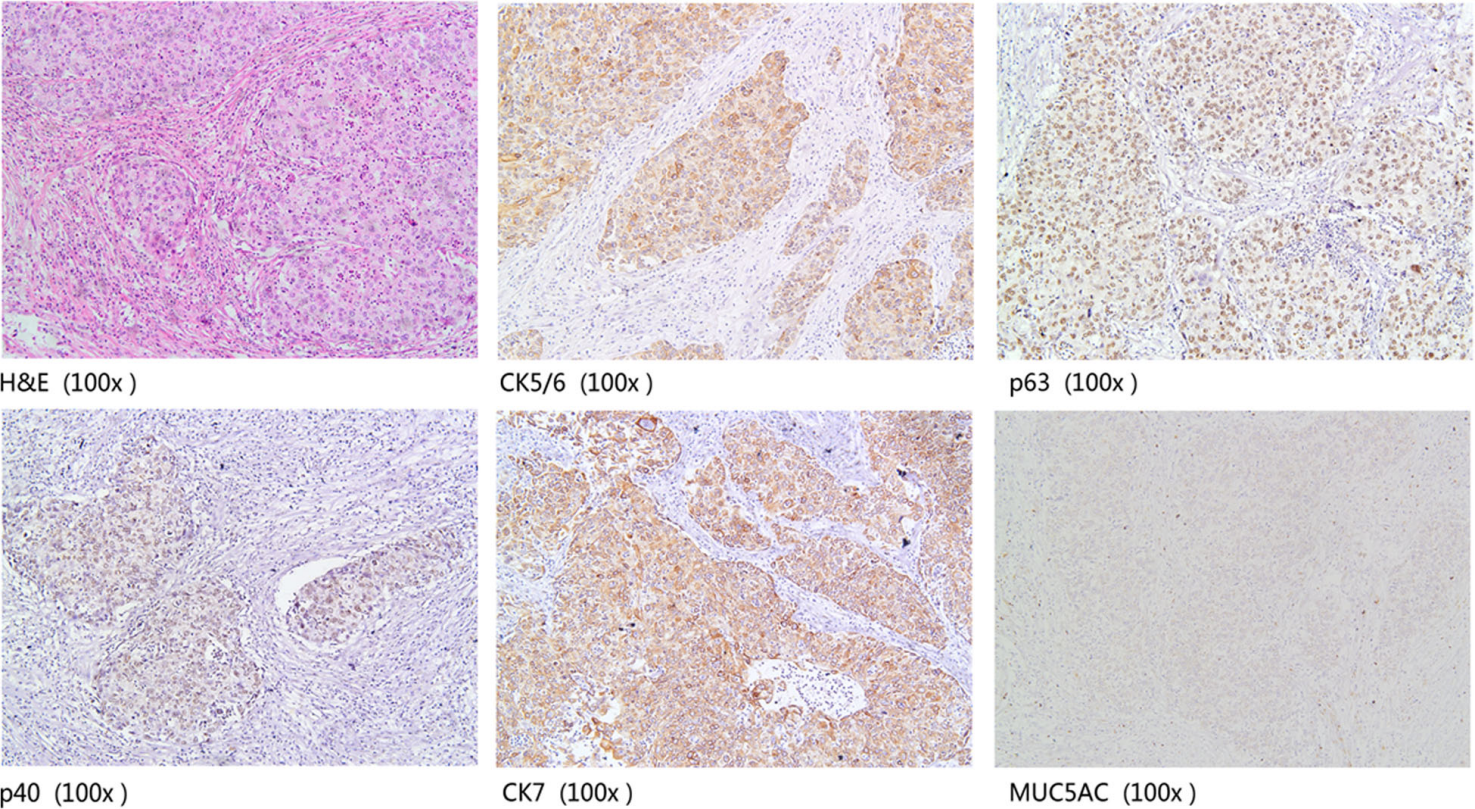
The expression of CK5/6, p63, p40, CK7, and MUC5AC in a case of poor-differentiated squamous cell carcinoma by IHC. **a** H&E; **b** CK5/6 positive staining; **c** p63 positive staining; **d** p40 positive staining; **e** CK7 positive staining; **f** MUC5AC negative staining (100×)

**Fig. 2:**
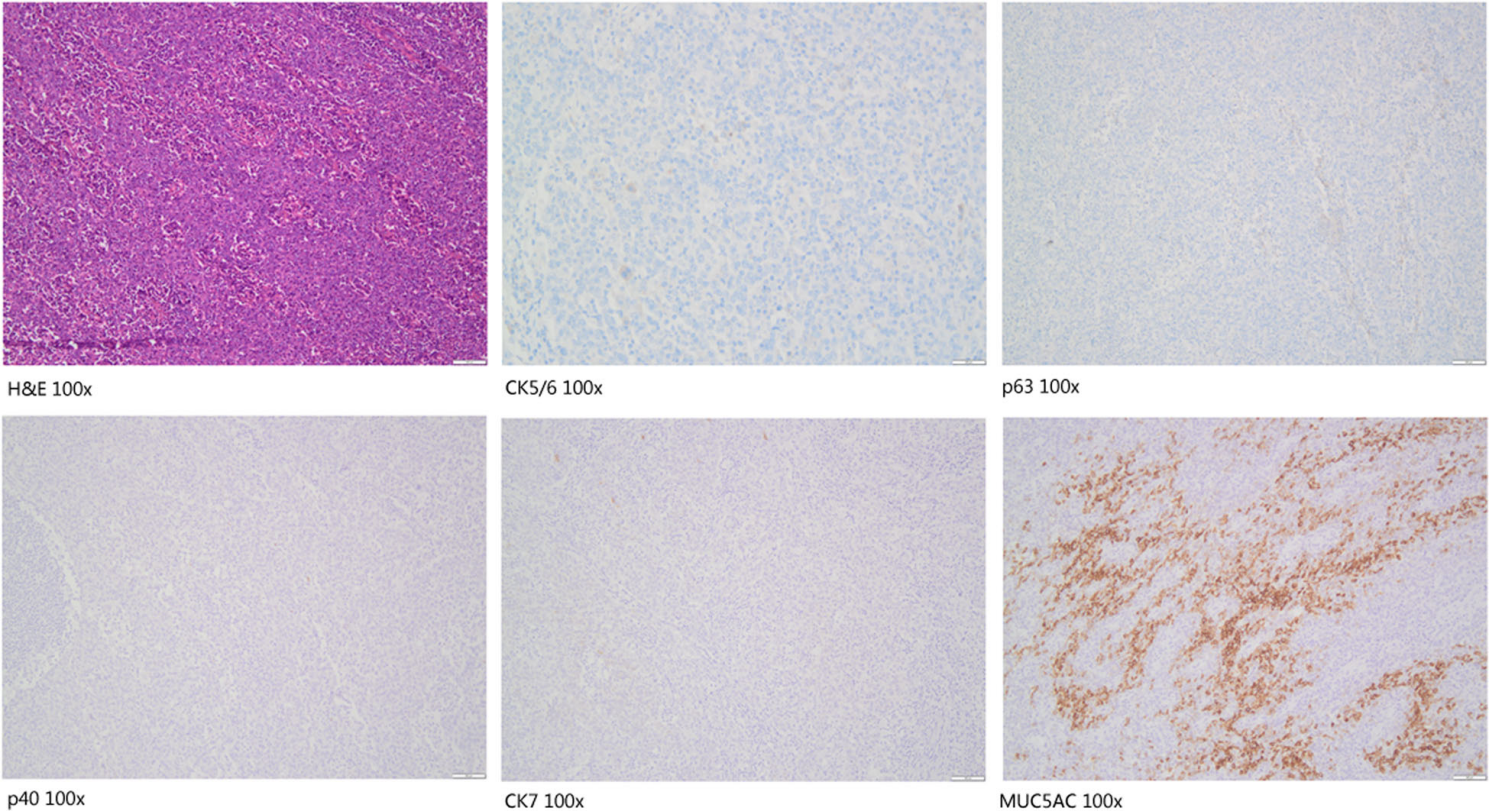
The expression of CK5/6, p63, p40, CK7, and MUC5AC in case of poor-differentiated adenocarcinoma (invasive stratified mucin-producing carcinoma, iSMILE) by IHC. **a** H&E; **b** CK5/6 negative staining; **c** p63 negative staining; **d** p40 negative staining; **e** CK7 negative staining; **f** MUC5AC positive staining (100×)

IHC for the five proteins was performed on 208 human primary cervical cancers, including 100 SCC and 108 AEC. As shown in Fig. 1 and Fig. 2, MUC5AC, CK5/6, and CK7 were mainly expressed in the cell membrane and cytoplasm, while p40 and p63 were mainly located in the nucleus.

We found that MUC5AC exhibited prominent immunoreactivity in the tumor cells of cervical AEC. MUC5AC and CK7 were detected in 81.48 and 82.41% of AEC cases compared to 9.9 and 49.50% of SCC cases. Besides, for AEC, the specificity of MUC5AC was much higher than that of CK7 (*P* < 0.05). Moreover, the sensitivity of CK5/6, p40, and p63 was 94.06, 85.15, and 89.11%, respectively, and the specificity was 77.78, 97.22, and 93.52% respectively in AEC (Table 3).

**Table 3:** Sensitivity and specificity of MUC5AC、CK5/6、CK7、P40、P63 in cervical squamous cell carcinoma and adenocarcinoma

Markers	squamous cell carcinomas(*n* = 101)	adenocarcinoma(*n* = 108)	Sensitivity(%)	specificity(%)
MUC5AC(+)	10	88	81.48	91.10
CK7(+)	50	89	82.41	50.50
CK5/6(+)	95	24	94.06	77.78
p40(+)	86	3	85.15	97.22
p63(+)	90	7	89.11	93.52
CK5/6(+)and p40(+)	83	2	82.18	98.15
CK5/6(+)and p63(+)	86	4	85.15	96.30
MUC5AC(+)and p40(−)	2	86	79.63	98.02
MUC5AC(+)and p63(−)	4	81	75.00	96.04
CK7(+)and p40(−)	6	86	79.63	94.06
CK7(+)and p63(−)	7	83	76.85	93.07

Through the combined detection of p40 or p63, we compared MUC5AC and CK7 again. We found that the sensitivity and specificity of MUC5AC in AEC combined with p40 or p63 were 79.63 and 75.00%, respectively; 98.02 and 96.04%, respectively. The sensitivity and specificity of CK7 combined with p40 or p63 were 79.63 and 76.85%; 94.06 and 93.07%, respectively (Table 3). The sensitivity of MUC5AC combined with p40 or p63 was similar to that of CK7, while the specificity was slightly higher than that of CK7.

### Correlation between MUC5AC expression and clinicopathological characteristics in cervical adenocarcinoma

**Fig. 3:**
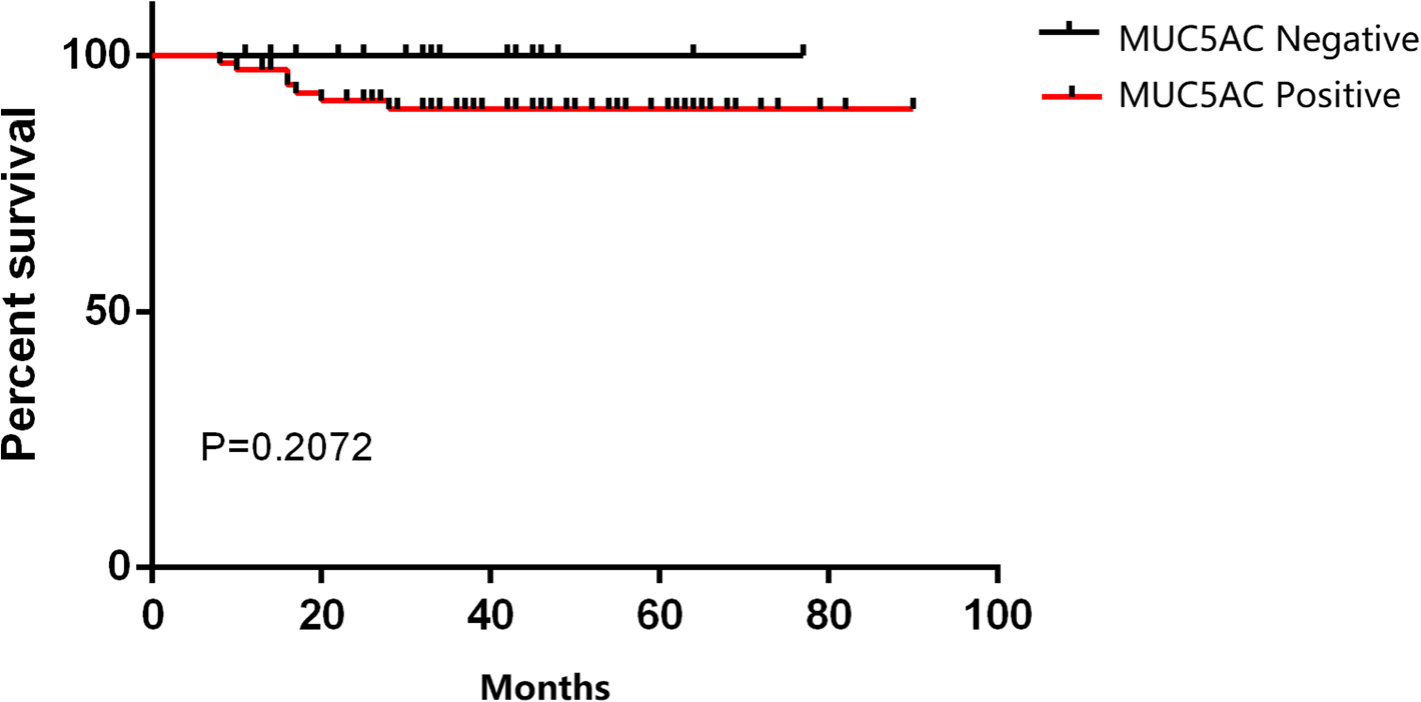
Survival analysis of MUC5AC expression in cervical adenocarcinoma

This study further analyzed the relationship between the expression of MUC5AC and clinicopathological features in cervical adenocarcinoma (Table 4). The expression of MUC5AC was correlated with the degree of tumor differentiation (*p* = 0.036). A lower degree of tumor differentiation was associated with a lower expression rate of MUC5AC. There was no significant correlation between the expression of MUC5AC protein and age, tumor size, depth of myometrial invasion, and lymph node metastasis (all *P* > 0.05). Kaplan Meier analysis revealed that the expression of MUC5AC protein had no significant effect on the prognosis of cervical adenocarcinoma patients (*P* > 0.05) as shown in Fig. 3.

**Table 4:** The correlation of MUC5AC and the clinical variants in the cervical adenocarcinoma

	The expression of MUC5AC		*χ 2* Value	*P* Value
	Positive	Negative		
**Age**				
≤ 45	44	6	2.622	0.105
v > 45	44	14		
**Size (cm)**				
< 4	63	12	1.581	0.209
≥ 4	22	8		
**Differentiation**				
Poor	25	11	4.388	0.036^*^
Well/Moderate	58	9		
**Infiltrate depth of mesenchyme**				
≤ 1/2	33	10	0.910	0.340
> 1/2	53	10		
**Lymph node metastasis**				
No	61	13	0.672	0.634
Yes	18	2

### Expression of MUC5AC and CK7 in cervical adenocarcinoma subtypes

We further detected the expression of MUC5AC in subtypes of AEC (Table 5). Among 52 cases of usual type cervical adenocarcinoma, 41 cases were MUC5AC positive, and 45 cases were CK7 positive, and there was no statistical difference (*P* = 0.448). In 9 cases of mucinous adenocarcinoma (NOS), the expression rate of MUC5AC and CK7 were both 88.88% (8/9). Moreover, 24 out of 26 cases of gastric mucinous adenocarcinoma expressed MUC5AC, and 23 of them were CK7 positive (*P* = 0.448). The positive rate of the MUC5AC in mucinous carcinoma (intestinal type), villous tubular adenocarcinoma, endometrioid adenocarcinoma, clear cell carcinoma, serous carcinoma, adenosquamous carcinoma and invasive stratified mucin-producing carcinoma (iSMILE) was 100, 66.67, 75, 100, 100, 50, and 100%, respectively. The expression rate of MUC5AC had no statistical difference among these subtypes (all *P* > 0.05).

**Table 5:** Expression of MUC5AC and CK7 in different adenocarcinoma subtypes

Subtypes	MUC5AC	CK7	*χ 2* Value	*P* Value
**Usual type**				
Positive	41	45	1.075	0.300
Negative	11	7		
**Mucinous adenocarcinoma**, **NOS**				
Positive	8	8	–	–
Negative	1	1		
**Gastric type**				
Positive	24	23	0.221	1.000
Negative	2	3		
**Intestinal type**				
Positive	1	1	–	–
Negative	0	0		
**Villous tubular adenocarcinoma**				
Positive	2	2	–	–
Negative	1	1		
**Endometrioid adenocarcinoma**				
Positive	3	1	1.400	0.559
Negative	4	6		
**Clear cell carcinoma**				
Positive	5	5	–	–
Negative	0	0		
**Serous carcinoma**				
Positive	1	1	–	–
Negative	0	0		
**iSMILE**				
Positive	2	1	1.333	1.000
Negative	0	1		
**Adenosquamous carcinoma**				
Positive	1	2	1.333	1.000
Negative	1	0		

*iSMILE* invasive stratified mucin-producing carcinoma

## Discussion and conclusions

Identification of previously unutilized, sensitive biomarkers is still a priority for improved differential diagnosis of cervical AEC and SCC. At present, CK5/6, p63, p40, and CK7 are the main biomarkers for differentiating cervical adenocarcinoma from squamous cell carcinoma.

CK5/6 is a kind of high molecular weight basal cell keratin (58kda and 56kda), which is mainly expressed in the basal cells of squamous epithelium and ductal epithelium, and some squamous epithelial germinal layer cells, myoepithelial cells, and mesothelial cells, but poorly expressed in glandular epithelial cells [8]. Some research results showed that CK5/6 has high sensitivity and specificity in the diagnosis of squamous cell carcinoma [8–10]. In contrast, other studies showed high sensitivity, but low specificity when diagnosing this type of tumor [11].

p63 is a member of the p53 family, a classical tumor suppressor gene family. It is located on chromosome 3q27–29. Filho et al showed good sensitivity when detecting squamous cell carcinoma with a positive rate of 92.6% [27]. Contrary, Kaufmann et al suggested that p63 could also be expressed in a small number of adenocarcinoma, basal cell carcinoma, and transitional epithelial carcinoma [9]. Moreover, p63 can also be used as a marker of myoepithelial cells and prostate basal cells. Therefore, p63 lacks absolute specificity for squamous differentiation.

p40 is a subtype of p63 protein expressed in squamous epithelial cells (including epidermis and hair follicles), urothelial cells, myoepithelial cells of the mammary gland, sweat gland and salivary gland and basal cells of the prostate, which are highly specific in labeling squamous epithelium [9]. Bishop et al showed that in 81 cases of squamous cell carcinoma of the lung and 237 cases of adenocarcinoma of the lung, the sensitivity and specificity of p63 were 100.00 and 69.20%, respectively. The sensitivity and specificity of p40 in the diagnosis of squamous cell carcinoma of the lung were 100 and 98%, respectively [20]. Therefore, p40 is considered as a highly specific and sensitive tumor biomarker of squamous epithelial origin [20].

In this study, we used immunohistochemistry to detect CK5/6, p63 and p40 in cervical squamous cell carcinoma and adenocarcinoma. The sensitivity of CK5/6, p40, and p63 was 94.06, 85.15, and 89.11%, respectively, and the specificity was 77.78, 97.22, and 93.52%, respectively. Moreover, the specificity of CK5/6 is slightly lower than that of p40 and p63. We also found that a combination of CK5/6 with p40 or p63 slightly decreased the sensitivity (82.18 and 85.15%), and increased the specificity (98.15 and 96.30%), which, in turn, increased the accuracy of diagnosing squamous cell carcinoma.

CK7 is a kind of low molecular weight keratin, mainly expressed in glandular epithelium and transitional epithelial cells of most normal tissues [20]. Many studies have found that CK7 is not only expressed in adenocarcinoma but also in squamous intraepithelial neoplasia, cervical squamous cell carcinoma, lung squamous cell carcinoma, and esophageal squamous cell carcinoma. Lee et al found a positive expression of CK7 in 66% (20 / 30) cases with SCC and 100% (25/25) cases with CINIII [20]. Furthermore, Yamada et al found that CK7 expression in esophageal squamous cell carcinoma, but also in I/IIA/IIB stage esophageal squamous cell carcinoma, suggest poor tumor differentiation, and thus can be used as an independent prognostic factor [28]. Our study showed that the positive rate of CK7 was 49% in cervical poorly differentiated squamous cell carcinoma, which further suggested that CK7 is not an ideal marker for differentiation between squamous cell carcinoma and adenocarcinoma.

Mucin is a high molecular weight glycosylated protein secreted by epithelial cells in the respiratory tract, gastrointestinal tract, and urogenital tract, which has an important role in the protection of epithelium, cell adhesion, signal transduction, immune activation, and inhibition. At present, at least 13 mucins have been found in the female reproductive system [29]. Riethdorf et al [30] and Albarracin et al [31] used immunohistochemistry methods to detect the expression of MUC5AC in different female reproductive system malignant tumors. They found that MUC5AC was highly expressed in cervical adenocarcinoma (75.6%, 31/41), and poorly expressed in endometrial adenocarcinoma (0.3%, 1/310). All of them were expressed in the primary ovarian mucinous tumor (100%, 32/32), but not in colon adenocarcinoma (0%, 0/10). Therefore, they concluded that MUC5AC could be used as an effective marker to distinguish the origin of pelvic tumors, and distinguish primary ovarian tumors and colorectal metastasis, as well as endometrial adenocarcinoma from cervical metastasis [30, 31]. In this study, we found positive expression of MUC5AC in 88/108 (81.48%) cases of cervical adenocarcinoma, and in 10/101 (9.90%) cases of squamous carcinoma, which was consistent with Riethdorf’s study [31]. The sensitivity of MUC5AC and CK7 to cervical adenocarcinoma was 81.48 and 82.41%, respectively; but the specificity of MUC5AC (91.10%) was much higher than that of CK7 (50.50%). Through the joint detection of p40 or p63, we compared MUC5AC and CK7 again, and found that the sensitivity and specificity of MUC5AC combined with p40 or p63 were 79.63 and 75.00% respectively, 98.02 and 96.04% respectively; the sensitivity and specificity of CK7 combined with p40 or p63 were 79.63 and 76.85%, 94.06 and 93.07% respectively. These results showed that the sensitivity of MUC5AC combined with p40 or p63 was similar to that of CK7, but the specificity was slightly higher than that of CK7. Therefore, MUC5AC is superior to CK7 in the diagnosis of cervical adenocarcinoma and squamous cell carcinoma.

Besides, we preliminarily detected the expression of MUC5AC in different types of cervical adenocarcinoma and found no significant difference. These data suggested that MUC5AC has no diagnostic significance in the classification of cervical adenocarcinoma. At the same time, we analyzed the relationship between the expression of MUC5AC and the prognosis of cervical adenocarcinoma, and the result revealed that MUC5AC was not related to the prognosis of cervical adenocarcinoma.

Overall, our observations strongly suggest that MUC5AC may be useful as a biomarker for differential diagnoses between squamous carcinoma and adenocarcinoma.

## Data Availability

Not applicable.

## Abbreviations

SCC: Squamous cell carcinoma
AEC: Adenocarcinoma of the cervix
CK: Cytokeratin
H&E: Hematoxylin & eosin
PBS: PHOSPHATE Buffered Saline
DAB: Diaminobenzidine
iSMILE: invasive stratified mucin-producing carcinoma
